# Feasibility of Forward Osmosis to Recover Textile Dyes Using Single Salts and Multicomponent Draw Solutions

**DOI:** 10.3390/membranes13120911

**Published:** 2023-12-18

**Authors:** Magdalena Cifuentes-Cabezas, Laura García-Suarez, José Luis Soler-Cabezas, Beatriz Cuartas-Uribe, Silvia Álvarez-Blanco, José Antonio Mendoza-Roca, María-Cinta Vincent-Vela

**Affiliations:** 1 Research Institute for Industrial, Radiophysical and Environmental Safety (ISIRYM), Universitat Politècnica de València, C/Camino de Vera s/n, 46022 Valencia, Spain; jsoca@isirym.upv.es (J.L.S.-C.); beacuau1@iqn.upv.es (B.C.-U.); sialvare@iqn.upv.es (S.Á.-B.); jamendoz@iqn.upv.es (J.A.M.-R.); mavinve@iqn.upv.es (M.-C.V.-V.); 2 Jeanologia S.L., Ronda de Guglielmo Marconi, 12, 46980 Valencia, Spain; 3 Department of Chemical and Nuclear Engineering, Universitat Politècnica de València, C/Camino de Vera s/n, 46022 Valencia, Spain

**Keywords:** forward osmosis, dye concentration, brine, textile wastewater

## Abstract

The textile industry generates large volumes of water characterized mainly by an intense color coming from dyes that are difficult to process due to their synthetic base and the presence of aromatic components. Due to the stricter regulation on the discharge of these effluents, in order to reduce dye waste before discharge into natural channels, alternatives are being sought to manage this wastewater. In this work, the concentration of dyes in simulated wastewater from the textile industry was studied by forward osmosis (with a cellulose triacetate CTA membrane), with the aim of concentrating the dye for its future recovery and reincorporation into the production process. Two dyes of different nature were evaluated to study the efficiency of the proposed process, using NaCl and reverse osmosis brine from a model seawater desalination solution as extraction solutions. It was observed that dye type (reactive or direct) and their charge influence the color rejection with the forward osmosis membrane used. It was able to concentrate the dyes in the feed solution up to approximately 55% with the reverse osmosis brine from the model seawater desalination solution. Finally, the results demonstrate that the FO process is a promising option for concentrating dyes present in wastewater from the textile industry in order to reuse them in the dyeing process.

## 1. Introduction

Due to rapid population growth, the global demand for freshwater has increased considerably. This generates the need to protect available freshwater resources, which is essential to ensure a sustainable water supply [1]. Therefore, there is a pressing need to investigate the reuse potential of treated wastewater for various industrial and drinking water reuse applications [2]. The textile industry is among the industries that generate the largest volumes of wastewater. It consumes around 77.5 × 10^9^ tons of water per year [3], thus contributing between 17–20% of global wastewater production [4]. These textile industry wastewaters are characterized by high pH, chemical oxygen demand (COD), total dissolved solids and intense color [5]. The intense color is given by different industrial dyes, which correspond to the largest group of environmental contaminants of organic compounds [6]. These dyes are not biodegradable; on the contrary, they are difficult to process due to their synthetic base and the presence of aromatic components in their chemical formula. Most of the dyes used are toxic and potentially carcinogenic to marine life and humans. Almost 20% of dyes are discharged into wastewater without additional treatment, which can also harm crops and agriculture [7]. The treatment of these wastewaters is a great challenge because they not only have different colorants but also a significant salt content. In light of this, the need for wastewater purification by eliminating waste from industrial dyes has increased, becoming one of the main environmental concerns [8]. For this reason, today, there are stricter regulations on the discharge of textile industry effluents in order to reduce dye residues in the treated wastewater before its release to natural streams [9].

Different methods for the treatment of textile wastewater have been reported in the literature, including both physical and chemical or biological processes [10,11,12] like degradation with bacteria [13,14]. Among them, membrane technology stands out because in recent years, it has been proven to be one of the most sustainable and most applied technologies in the treatment of textile wastewater. On the other hand, membrane technology has demonstrated greater dye recovery capacity and has been recommended as one of the best available techniques for cleaner production in the textile industry [15]. This is mainly because it is one of the best methods in terms of novelty, efficiency and purity for obtaining the desired water quality for reuse.

Membrane pore sizes are narrow enough to retain the dye compounds [16]. Due to this, many studies have been published regarding the removal of dyes using membrane technologies, mainly those that use pressure as a driving force, classifying them into microfiltration (MF), ultrafiltration (UF), nanofiltration (NF) and reverse osmosis (RO). MF is mainly used to remove colloidal dyes due to its larger pore size or as pre-treatment to other membrane processes [17]. UF membranes are used to recover higher molecular weight substances, such as auxiliary chemicals or some other dyes [18,19,20]. NF membranes can separate organic compounds, divalent salts and dyes present in wastewater [21,22,23], achieving, alike the UF in some cases, a permeate that could be reused in the rinsing and washing processes of the textile industry; meanwhile, RO membranes can retain all mineral salts and hydrolyzed reactive dyes [24]. Several studies propose combinations of UF and NF membranes that achieve the water quality required for water reuse applications [25]. They entail the cost of implementing two membrane processes where sometimes pressures over 20 bar are necessary for the separation process and need to deal with high fouling problems. However, there are membrane processes driven by an osmotic gradient, such as forward osmosis (FO). Unlike processes driven by external pressure, this technology is driven by the difference in the concentration of osmotic solutes between a concentrated draw solution (DS) and a less concentrated feed solution (FS). This implies a low energy consumption and less membrane fouling compared to pressure-driven membrane processes. FO allows for the achievement of considerable water recovery, with a high adaptability for complex liquid streams [15]. FO is a promising technology for the treatment of textile wastewater, since dyes are retained by the FO membranes and water permeates through them. In this way, dyes can be concentrated for their reuse. At the same time, salt passage from the draw to the feed solution occurs due to the concentration difference (Fick’s law). However, this named reverse salt flux is not a problem for the dyeing process, which requires salts to fix the color on the fabric. Li et al. [26] presented a novel three-stage FO proposal using a symmetrical FO membrane developed by them and sodium polyacrylate (PAA-Na) as the DS. They achieved the simultaneous desalination and concentration of synthetic textile wastewater. Han et al. [27] also obtained good results when testing a thin-film composite FO membrane (TFC) manufactured in the laboratory, with 2 M NaCl as the DS. They achieved a dye rejection of 99.9% with wastewater from synthetic textiles containing multiple textile dyes, inorganic salts and organic additives.

In recent years, desalination using reverse osmosis has also been in the spotlight, not only because it is a reliable source of fresh water but also because of the concentrated saline solution generated (reverse osmosis brine from seawater desalination), which is difficult to manage. Therefore, companies must not only comply with the requirements for the reuse of desalinated water but also with brine discharge regulations [28,29,30]. This is where this reverse osmosis brine from seawater desalination comes into play as an option to consider as a DS candidate. This is because it possesses the osmotic potential to concentrate dyes in the FS and simultaneously achieve its dilution in the DS. In other works [31], the diluted DS of the FO is pumped into a RO system to obtain a high-quality water (RO permeate) and a brine that can be used again as a DS in the FO process.

In this work, the concentration of dyes in a simulated textile industry stream was studied using forward osmosis with both a NaCl solution and a reverse osmosis brine from a seawater desalination model solution. The objective was to achieve a final dye concentration in the feed suitable for its reincorporation into the production process in the future. For this purpose, different dyes of different nature were used to evaluate the efficiency of the proposed process. The novelty of this work is to propose the use of two residual streams, textile wastewater and reverse osmosis brine from seawater desalination, in a FO process to concentrate dyes for their further reuse in the textile industry while diluting the seawater desalination brine. To the best of our knowledge, there is a shortage of studies dealing with dye concentration by osmotically driven processes. Our study has the advantage of using seawater reverse osmosis desalination brine (considered as a waste) as the draw solution in the forward osmosis process and diluting at the same time this brine, thus making it easier to manage by having a lower salt concentration. The concentrated dye solution can be reused in the dyeing process, contributing to the circular economy. Unlike the authors, for example, Yasmeen et al. [5] used sodium dodecyl sulfate solution as the draw solution in textile dye concentration by forward osmosis. This work aims to encourage further study of the use of these waste streams, since both are currently difficult to manage for industries.

## 2. Materials and Methods

### 2.1. Draw and Feed Solutions

In the FO tests, only model solutions (MS) were used for both the DS and FS. NaCl (1 M, 58.44 g·L^−1^) and reverse osmosis brine from a seawater desalination model solution (SBMS) were used as the DS. The SBMS was composed of 58.44 g·L^−1^ NaCl, 8.50 g·L^−1^ Na_2_SO_4_ and 25.76 g·L^−1^ MgCl_2_·6H_2_O. For the FS, two model solutions (MS) were used, varying only the dyes, with a composition of 0.05 g·L^−1^ dye + 0.5 g·L^−1^ Na_2_SO_4_ + 0.5 g·L^−1^ NaCl. Dystar supplied both dyes (dye characteristics are presented in Table 1) and PanReac AppliChem (Spain) supplied all reagents, which were all over 98% of purity. All of the FS and DS solutions used had a pH range between 6.18 and 6.72. The concentration of dyes and salts used in the feed solutions mimicked those of a real textile industry. In the literature, there is a wide range of dye concentrations reported for the composition of synthetic wastewaters. Aouni et al. [32] worked with a dye concentration of 0.6 g·L^−1^ (a high value), while Keskinkan et al. [33] used a concentration of 0.011 g·L^−1^ (a low value) g·L^−1^. Muda et al. [34] used a dye concentration of 0.05 g·L^−1^, the same as used in this work. Yaseen et al. [35] reported a NaCl concentration of 300 m g·L^−1^, a similar value to the 500 m g·L^−1^ used in this paper. In addition, they reported a sulphates concentration of 387 m g·L^−1^ (in Spain), very similar to the 338.15 m g·L^−1^ that contributes 0.5 g·L^−1^ Na_2_SO_4_ in this article.

### 2.2. Forward Osmosis Laboratory-Scale Plant

**Figure 1:**
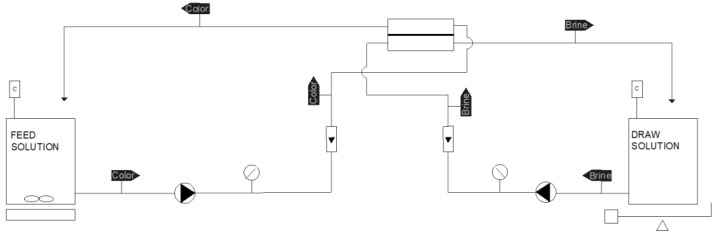
Laboratory forward osmosis plant (diagram).

The forward osmosis laboratory-scale plant is presented in Figure 1. The main part is a forward osmosis flat-sheet membrane module (model CF042, Sterlitech, Auburn, WA, USA). Two PVC 5 L tanks house the FS and DS solutions, respectively. To measure the pressure and flow of both the FS and DS, two manometers (from NUOVOFIMA, Invorio, Italy) and two flowmeters (2300 model, from Tecfluid S.A., Barcelona, Spain) were used, respectively. The DS tank was continuously stirred with a magnetic stirrer. Two peristaltic pumps (from Heidolph (Schwabach, Germany), Pumpdrive 5106 model) impelled both the FS and DS solutions, respectively. With the aim of recording the conductivity of both the FS and DS tanks, two conductivity meters (from Omega Engineering, model CDH-SD1, Mancherter, UK) were used. In order to measure the change in the mass of the DS tank, a precision scale (from Kern (Balingen, Germany), model “PKP” with RS-232 port), a personal computer with a RS-232 to USB cable and a data logger software (Kern Balance Connection, version SCD-4.02, from Kern) were used. To measure the pH variations, a pH meter model “HD2305.0” from Delta-Ohm was employed.

The FO membrane tested was a cellulose triacetate (CTA) flat-sheet membrane (FTSH2O from Fluid Technology Solutions, Albany, OR, USA) of 0.0042 m^2^ active area. The membrane has a contact angle of 68.1 ± 1° (active layer), a Zeta potential of −12.8 ± 1.18 mV [36] and a pore diameter of 0.307 ± 0.003 nm [37]. The membrane module CF042-FO was provided by Sterlitech, Auburn, WA, USA. The membrane was selected due to the intrinsic characteristics of this material. Its efficient use in wastewater treatment has been demonstrated due to its high water permeability, effectiveness in rejecting dyes and contaminants, and antifouling properties. In addition, this membrane is manufactured at a large industrial scale [36].

### 2.3. Water Flux Variation over Time

In order to determine the quantity of water that passes through the membrane, the draw solution tank was weighted during the test with a digital scale connected to a laptop. 

During operation, the volume of the draw solution tank increased as the volume of the feed solution tank decreased due to the aforementioned pass of water through the membrane. (See the following equation (Equation (1)).

(1) $$Jw=\frac{\left(m_{draw}^{final}-m_{draw}^{initial}\right)/\rho_{water}}{A_{m}\cdot t}$$

where ‘*m*’ is mass, ‘*ρ*’ is density, ‘*Am*‘ is the active membrane area and ‘*t*’ is time. Water flux was corrected with temperature, taking into account the following equation (Equation (2)):

(2) $$J_{w1}\cdot \mu \left(T_{1}\right)=J_{w2}\cdot \mu \left(T_{2}\right)$$

where ‘*µ*’ is viscosity and ‘*T*’ temperature.

The reverse salt flux (*Js*) was measured, as can be seen in the following equation (Equation (3)):

(3) $$J_{s}=\frac{V_{feed}^{final}\cdot C_{feed}^{final}-V_{feed}^{initial}\cdot C_{feed}^{initial}}{A_{m}\cdot t}$$

where ‘*V*’ is volume, ‘*C*’ is concentration, ‘*Am*’ is the active area of the membrane and ‘*t*’ is time.

### 2.4. Methodology

Before each forward osmosis test, the draw and feed tank were filled with the DS and FS, respectively. The pumps of the FS and DS were switched on and the flow rates were set (30 L·h^−1^ for both feed and draw circuits). Then, the conductivity meters were switched on, and the data logging was started. Also, the data logging of the values of weight was switched on. The two manometers should show values close to zero, as forward osmosis is a non-pressure-driven process. When the test was finished, data logging was stopped and the pumps were switched off.

As seen in other previous works [31,38,39], the most suitable salts to be used as the DS, in order to dewater textile wastewater, are NaCl, MgCl_2_ and Na_2_SO_4_. Dutta et al. [31] proved that Na_2_SO_4_ is a suitable draw solute for textile dewatering; Dutta et al. [38] used MgCl_2_ as the draw salt for color removal from dye solution in a forward osmosis process, while Wu et al. [39] tested a novel conductive carbon-based FO (CPFO) membrane with NaCl as the DS. Hongmei Yuan et al. [40] used NaCl, Na_2_SO_4_ and MgCl_2_ salts as the draw solution, as the authors have done in this article. Therefore, these salts were evaluated separately in batch tests for 1 h. 

The conductivity measure was easier (using a conductivity meter) and faster to perform than the concentration measure. Then, three calibration curves (one for each salt) were performed in order to measure the salt concentration of the samples. Three forward osmosis tests were performed, using concentrations of 1 M in the DS and deionized water as the FS. 

Once the salt was selected (NaCl), in order to evaluate the membrane performer, two more tests were performed, using 2M and 3M NaCl as the DS, respectively, and deionized water as the FS. Then, the tests for the dye concentration were performed. 

In Table 2, the different tests performed with the different DS and FS are depicted. A first study was made to test the feasibility of dye concentration using the FTSH2O membrane with two different DSs. For that purpose, test 1 and test 2 were carried out with the same FS (MS1), varying the DS, and using NaCl and SBMS. Finally, the last test (test 3) was performed using the best DS (SBMS) and MS2 to check the efficiency of the DS when using a different dye. The three tests were carried out under the same flow rate conditions indicated above and lasted 48 h. 

### 2.5. Cleaning Procedure

After every test, the membrane was cleaned. In the case of the characterization tests, the plant was operated using osmotized water (conductivity < 40 µS·cm^−1^) as the FS and tap water as the DS for approximately 5 min. Subsequently, the conductivity of the two solutions was measured. If the conductivity of the DS was below 1300 µS·cm^−1^ and the conductivity of the FS was around 60 µS·cm^−1^, the plant was stopped, and the two solutions were replaced by deionized water; otherwise, the cycle was repeated. Once the two solutions had been replaced by osmotized water and after 5 min of operation, the conductivity was measured in both sides of the membrane, and if a conductivity value lower than 60 µS·cm^−1^ was achieved, the cleaning was considered to be completed. Otherwise, the solutions were renewed again, and after 5 min, the conductivity was measured again. For the dye concentration tests, a similar process was carried out. However, first, both solutions, the FS and DS, were replaced with tap water, renewing them approximately every 5 min until no dye was visually appreciated.

### 2.6. Analytical Methods

Two conductivity meters recorded the value of conductivity over time in the tanks. During operation, the conductivity of the DS decreased as the conductivity of the FS increased due to the volume reduction in the feed solution tank (concentration effect) and the rise in the volume of the draw solution tank (dilution effect).

In order to determine the color of the samples, the “*FZ*” parameter was measured. This parameter is the standard value to measure in the dye industry [41], and it is a weighted average calculated as in the following equation (Equation (4)):

(4) $$FZ=\frac{\lambda_{436}^{2}+\lambda_{525}^{2}+\lambda_{620}^{2}}{\lambda_{436}+\lambda_{525}+\lambda_{620}}$$

In order to determine the dye concentration achieved through the forward osmosis process, samples of the FS at the beginning and at the end of the tests were taken. Then, the color of the samples was determined using a spectrophotometer, measuring the absorbances at three different specific wavelengths (436, 525 and 620 nm) and applying the aforementioned FZ formula. The absorbances were higher when the dye concentration was higher. For ion determination, a “Nova 30 A” spectrophotometer from “Spectroquant” was employed, using kits from Merck, namely: 1.00815.001 (sodium, Na^+^), 1.14548.001 (sulphate, SO_4_^2−^), 1.14730.001 (chloride, Cl^−^), and 1.00815.001 (magnesium, Mg^2+^). On the other hand, a spectrophotometer model “DR600” from “Hach Lange” was used to measure the absorbances (and then the color) of the samples.

## 3. Results

### 3.1. Membrane Characterization

From the studies of the different salts (NaCl, MgCl_2_ and Na_2_SO_4_), the DS with NaCl was selected (Figure S1), considering both the values of water fluxes and salt fluxes (Jw and Js), as well as the price. 

**Figure 2:**
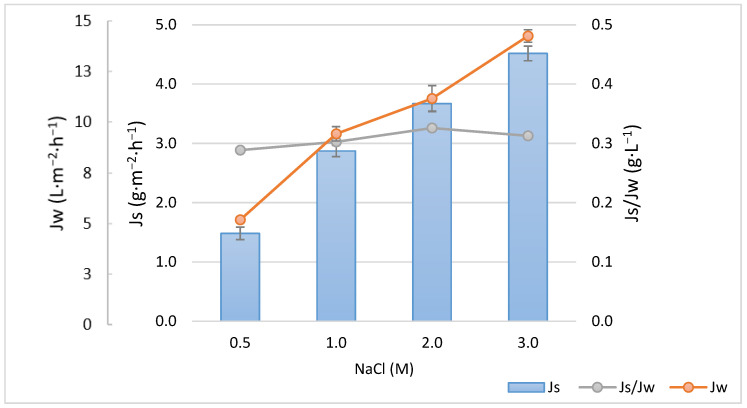
Membrane characterization: permeate flux (Jw) and reverse salt flux (Js) and specific reverse salt flux (Js/Jw) as a function of NaCl concentration, with FS and DS flow rates of 30 L·h^−1^.

The relationship between the water permeate flux (Jw), the reverse salt flux (Js) and the specific reverse salt flux (Js/Jw) of the membrane as a function of NaCl concentration, with deionized water as the FS, is presented in Figure 2. It shows the behavior of Jw with an increasing concentration of NaCl in the DS. A linear trend in Jw is observed as the concentration increases. This trend suggests that there is no limitation on membrane permeability with increasing NaCl concentrations, which means that the dilutive external concentration polarization (ECP) on the permeate side of the membrane has no significant effect on the osmotic driving force [42]. Concentrative ECP is caused by the solutes retained by the membrane, increasing their concentration on the membrane surface. This phenomenon contributes to a decrease in the osmotic pressure difference between both sides of the membrane and, consequently, the water flux decreases. It can be seen in Figure 2 that Js also increases linearly with NaCl concentration. On the other hand, the constant ratio of Js/Jw around 0.31 ± 0.1 g·L^−1^ suggests that at a higher concentration of NaCl, the properties of the inner membrane remain constant.

Following the methodology presented by Cifuentes-Cabezas et al. [43] and with the Matlab tool, the intrinsic characteristic parameters, which completely describe the performance of the membrane, were determined. These correspond to water permeability (Aw), salt permeability (Bs) and a structural parameter (S) related to the internal polarization modulus Ks. The values obtained were Aw= 0.78 ± 0.03 L·m^−2^·h^−1^·bar^−1^, Bs = 0.2 ± 0.01 L·m^−2^·h^−1^ and S = 649 ± 7.4 µm (Ks = 2.87·105 s∙m^−1^). These values are similar to those found in the literature [36,44] for CTA membranes.

### 3.2. Dye Concentration Tests

The results obtained in the dye concentration tests are presented below. In the first instance, two DSs (NaCl 1M and SBMS) are evaluated to analyze their efficiency when concentrating on a specific type of dye. Then, with the best DS, the concentration efficiency of the process is evaluated using a different commercial dye.

#### 3.2.1. Influence of the Type of DS on the Concentration Efficiency 

**Figure 3:**
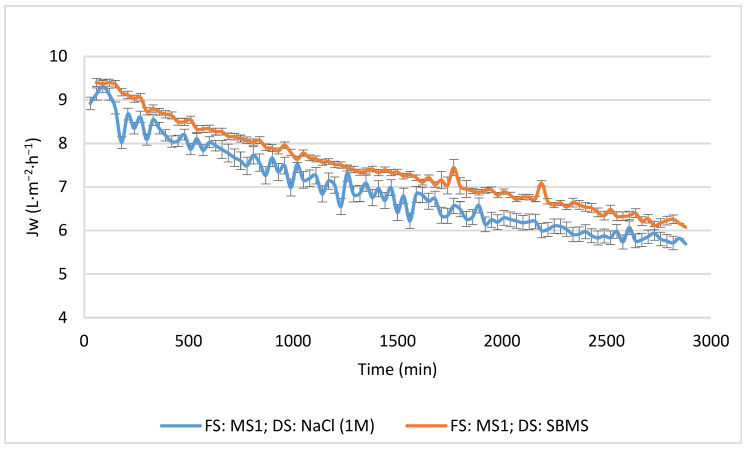
Membrane permeate flux during time. Comparison between two draw solutions, NaCl (test 1) and SBMS (test 2), with FS and DS flow rates of 30 L·h^−1^.

Figure 3 shows the comparison between NaCl and SBMS, with MS1 as the FS, in terms of the permeate flux. It can clearly be seen how both DSs presented a similar trend, starting with a permeate flux of over 9 L·m^−2^·h^−1^ and declining at approximately 34–35% after 48 h. The decrease in the permeate flux is generated mainly due to a decrease in the driving force in the FO process. This could be due to increasing salinity on the FS side and dilution on the DS side. Another factor to consider is the mode of operation. In this case, it was the active layer of the membrane facing FS (AL-FS). In this configuration, concentration polarization occurs within the porous FO support layer, called internal concentration polarization (ICP), which causes a decrease in the permeate flux [45,46].

On the other hand, the decision was made to work in counter current mode, as it has been reported to increase driving force compared to parallel operation [47]. The observed large decrease in the permeate flux could be attributed to ECP and ICP. As in these cases, the FS corresponds to a more complex (presence of salts) FS than water, and concentrative ECP can occur, further reducing the driving force and hence the flux. An accumulation of dye present in the feed on the membrane surface could also occur. However, the FS presents relative low salt concentration, and the DS on the support layer will also be subjected to dilutive ICP due to a back diffusion of salts from the DS toward the FS which increases the effect of ICP [48]. The decrease in Jw could also be due to the diffusion of salts from the DS to the FS, forming cake-enhanced osmotic pressure (CEOP). This is a resistance generated by the accumulation of salts near the active surface of the membrane, together with the rest of the solutes present in the FS, which, according to [49], is one of the main contributors to the overall decline in Jw. On the other hand, if CEOP forms, it could lead to another phenomenon called capillary force resistance (CFR). CFR manifests as a loss of pressure from the FS at the membrane interface, resulting in a decrease in the flow of water from the feed solution toward the membrane surface [36].

It is important to consider that the permeate flux, when using deionized water as the FS and 1 M NaCl as the DS, was 9.49 L·m^−2^·h^−1^, so there was no significant variation in the initial permeate flux when the dye was added to the FS. Therefore, as other studies indicate that the dyes contribute to membrane fouling by forming a gel layer on the surface of the FO membrane, this also evidences the decrease in the permeate flux over time.

Regarding the values of Jw, the SBMS always promotes a higher and smoother permeate flux, being 10% higher than that obtained with 1M NaCl. Although NaCl is the salt by excellence to prepare a DS, it achieves a higher permeate flux than divalent salts at similar osmotic pressures, as the presence of the other salts (multicomponent or mixed DS) favors the osmotic pressure (driving force) [50,51,52]. Thus, Hamdan et al. [50] concluded that the ternary solution of MgCl_2_ + NaCl yields a higher osmotic pressure than that of the MgCl_2_ solution. Holloway et al. [52] and Ibrar et al. [51] reported that solution diffusion mechanisms rather than electrostatic interactions govern the water transport. For the ions, transport from the DS to the FS is higher for monovalent ions due to their diffusivity and mobility. It entails a quicker increase in ion concentration in the FS, reducing the osmotic pressure difference between both sides of the membrane and decreasing the water flux.

Therefore, a greater passage of water through the membrane is achieved. Considering then that using reverse osmosis brine from a seawater desalination model solution as the DS, apart from providing greater osmotic potential, has the advantage of also dealing with a concentrate that is difficult to treat, it is of greater interest for use as a DS. As Chia et al. [53] rightly point out, the main concern in desalination is brine. This is because its discharge into the sea can affect the seabed due to its different temperature, salinity and density compared to seawater and the possible toxicity when sinking, affecting the communities that inhabit the seabed. Therefore, the incorporation and use of reverse osmosis brine from seawater desalination is, in addition to a saving in reagent utility costs, beneficial to the environment.

The potential of using brines as a DS has already been presented in other works. One such case is the use of hypersaline brine extracted from a potential CO_2_ sequestration site as the DS to concentrate industrial wastewater [54]. The authors observed a higher flux of brine compared to MgSO_4_ (20%) when used as the DS.

**Figure 4:**
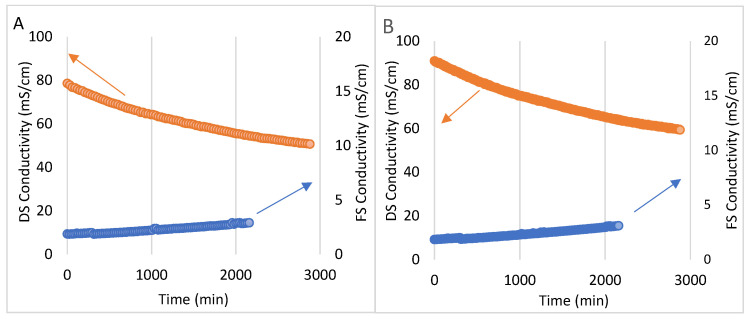
Conductivity of both draw solution (DS, in orange) and feed solution (FS, in blue) for test 1 (**A**) and test 2 (**B**), with FS and DS flow rates of 30 L·h^−1^. The arrows indicate which axis it corresponds to.

As can be seen in Figure 4, the initial conductivity of the SBMS (Figure 4B) is higher (90.1 mS·cm^−1^) than the one corresponding to NaCl (77.8 mS·cm^−1^) in test 1 (Figure 4A). However, both DSs decrease its conductivity by around 34% and provide sufficient osmotic pressure to increase the FS conductivity by 55% and 65%, for test 1 and test 2, respectively. These changes in the conductivity in both tests are attributed to the accumulation of feed solutes and the reverse diffusion of the DS salts, which will result in a drop in the driving force up to a certain point, which in turn is directly related to the decline in Jw, as observed in Figure 3. This increase in FS conductivity is also reflected in the concentration of the different parameters analyzed (Table 3). In the same way, the dilution of the same parameters in the DS is confirmed.

Table 3 shows how SBMS leads to a higher Cl^−^ ion concentration in the FS. All of the ions analyzed in the FS presented variation concerning the DS used, achieving higher concentration rates when SBMS was used as the DS (test 2). For example, for Cl^−^, both test 1 and test 2 started with a concentration close to 0.37 ± 0.01 g·L^−1^, reaching a value of 0.66 g·L^−1^ vs. 0.71 g·L^−1^ at the end of the test, respectively. This is equivalent to an increase in the concentration of the Cl^−^ ion of 87% (test 2) and 78% (test 1) in the FS. Regarding the DS, it can be seen in Table 3 that there was a decrease in the concentration of the ions, observing for all of the ions a similar decrease in their concentration in test 1. For example, a decrease in Na^+^ concentration by 54% (from 30.9 g·L^−1^ to 14.2 g·L^−1^) and 49% (from 32.7 g·L^−1^ to 16.6 g·L^−1^) is observed in test 1 and test 2, respectively. Despite, the conductivity values of the final FS (concentrate), using NaCl and SBMS, increased by 54.8% and 64.4%, respectively (Figure 4). The color values measured by the FZ value (Table 3) did not present as a high variation between the tests as the conductivity, increasing on average by 54% ± 1 (average of test 1 and test 2), with the concentration of dyes in test 1 being approximately 55% (the highest). Although analyzing the percentage values of conductivity reveals an increase, a variation is observed between the use of NaCl and SBMS, and it is a fact that the conductivity values do not differ that much. This is because they are relatively low values, so a variation between them generates a higher impact. Then, the FS concentrated using NaCl as DS changes from 1.88 mS·cm^−1^ to 2.91 mS·cm^−1^, while the FS concentrated by SBMS changes from 1.87 mS·cm^−1^ to 3.09 mS·cm^−1^.

After the tests, the membrane was able to recover its initial permeability. This supports the decision to work in ALFS mode. Although working in ALDS mode makes it possible to obtain a higher permeate flux with a less severe concentration polarization effect, working in ALFS mode makes it easier to remove fouling from the dense active layer than from the porous support layer [55].

#### 3.2.2. Influence of the Type of Dye on the Concentration Efficiency

The two selected dyes (Table 1) were chosen because they belong to two of the most widely used groups of dyes in the textile industry: reactive dyes and direct dyes [56,57]. These groups have the lowest degree of fixation to the fabric. Therefore, a considerable amount of dye is lost in the effluent without fully adhering to the fiber [58]. In this case, since the two model solutions were prepared with the same reagents and concentrations, by only changing the type of dye, it will be the dyes that will influence the permeate flux.

**Figure 5:**
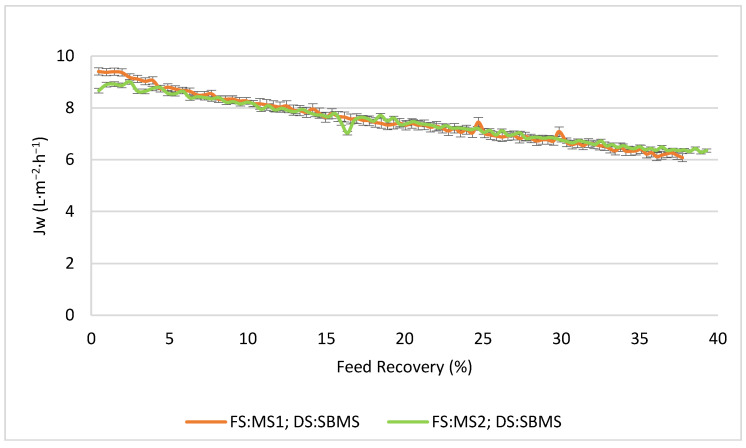
Permeate flux as a function of feed water recovery rate. Comparison between two commercial dyes, Remazol turquoise G-133% (test 2) and Sirius Blue K-CFN (test 3), with FS and DS flow rates of 30 L·h^−1^.

Figure 5 presents the feed water recovery rate (%), calculated based on a previous study [27], using the following equation (Equation (5)):

(5) $$Feed\;recovery\;(\%)=\frac{∆V}{V_{f,i}}\cdot 100$$

where *V_f,i_* (L) is the initial volume of the feed solution and Δ*V* (L) is the permeated water over a predetermined time Δ*t* (h). As both tests were carried out for 48 h, the idea is to analyze if it is possible to reach 40% of the recovery rate (maximum value, conditioned by the volume of the test vessel). It can be seen that almost 40% of the recovery rate is achieved with both MS, achieving 37.7% with MS1 (test 2) and 39.3% with MS2 (test 3).

Regarding the Jw, the values obtained with both dyes were practically the same, except at the beginning, when test 2 achieved a slightly higher value (9.4 vs. 8.6 L·m^−2^·h^−1^); after this, both tests managed to perform in a similar way. However, both tests showed a relatively constant decline. Initially, the decrease was more abrupt, losing 23% compared to the initial flux during the first 30 h of operation. Then, a slighter decrease (4–6%) was observed that becomes almost constant until the end. The first reduction in flux is the result of the deposition of dye molecules on the membrane surface, and then, the reduction gradually stabilizes, with the flux decrease being smoother and slower. Furthermore, it is necessary to highlight that in both tests 2 and 3, the ion concentrations were higher than that of test 1, which agrees with the Jw results observed.

The similar Jw obtained with the different dyes used (Remazol turquoise G-133% and Sirius Blue K-CFN) in the tests with MS1 and MS2 may be related to the molecular weight of the substances, as Makki et al. [59] observed in a study on the concentration of different dyes (Disperse Blue and Reactive Red) by means of FO also using a CTA membrane. They observed that for two types of FS with the same concentration, the permeate flux is limited by the molecular weight of the substances that compose it. As noted above, the only variation between MS1 and MS2 was the type of dye used. Then, since the molecular weights of the dyes were of the same order of magnitude (1079.55 g·mol^−1^ and 1098.93 g·mol^−1^, for the dyes present in MS1 and MS2, respectively; Table 1), the Jw also was of the same order of magnitude. Although studies varying dye concentration have not been performed, other authors have concluded that membrane fouling in the FO mode is more closely related to dye chemistry and molecule weight but is less sensitive to dye concentration [27]. They observed that a variation from 100 ppm of INCA dye to 300 ppm only presented a slight variation in the Jw. However, Jw did not change when the dye concentration increased up to 500 ppm. This suggests that the membrane can be saturated at a low dye concentration, without subsequently affecting the Jw. Therefore, it could be inferred that the same trend would occur when having higher dye concentrations.

Other FO studies carried out with reactive dyes (Remazol Brilliant Orange FR and Remazol Blue BR) reported greater variation in the permeate flux over time with the Orange FR reactive dye than with Blue BR [60]. This may be attributed to the chromophore group of the dye. Thus, although both are reactive dyes, Orange FR has an azo chemical structure (N = N), while Remazol Blue BR is of the anthraquinone type [6]. The behavior of Jw observed in the study carried out with the aforementioned Orange FR dye is similar to the behavior observed in our study. This may be because it has a similar azo structure to Sirius Blue K-CFN, which was used in this study. This implies that the Jw can be influenced both by the molecular weight of the substance and by the different chromophore group.

Regarding the chemistry of the dyes, although both are of different types, one is characterized by a phthalocyanine class with sulfonic group complexed with copper [61,62] and the other by an azo group [63]; both are anionic dyes. Therefore, the FTSH2O membrane has a negatively charged surface [64,65] in the pH range of the FS (pH of 6.65 and 6.97 for MS1 and MS2, respectively), with no adsorption of the dyes occurring on the membrane surface. Therefore, this facilitates the concentration and recovery of the dyes as well as membrane cleaning. Other authors point out something similar in NF membranes: negatively charged membranes increase the electrostatic repulsion of anionic dye compounds. Then, the sulfonate groups interact strongly with the negatively charged membrane. They attribute this phenomenon to high color retention and better chloride retention which affect the observed conductivity retention rates [32]. It has been reported that, in general, the rejection of ionic components by the CTA FO membrane may be due to the sieve effect and the electrostatic interaction between the dissolved substance and the negatively charged active layer of the CTA membrane [66].

As can be seen in Table 4, the color of MS2 increased by 50%, 5% less than what was achieved with MS1. On the other hand, the reverse passage of salts (Js) did not present great variation with respect to that obtained in the characterization. This was confirmed with the analyzed ions, with their concentration corresponding to the decrease and increase in the volume of both solutions (FS and DS).

Regarding cleaning, in all tests, optimal cleaning of the plant was achieved, reaching conductivity on the DS side below 1300 µS·cm^−1^ and around 60 µS·cm^−1^ for the FS side. Curiously, MS1 with Remazol turquoise blue G dye was the most difficult to remove from the plant (membrane and piping system). This may be mainly due to the binding that the dye achieves with the membrane. Although, as mentioned above, the negative charge of the membrane and the pH of the solutions used did not favor the possibility of adsorption of the dyes on the membrane, which could also occur. It has been reported that reactive dyes (such as the one used in MS1), unlike direct dyes, can create a chemical bond with the fiber [67]. This can create a stronger bond with the membrane, making it more difficult to clean. On the other hand, reactive dyes also have a greater affinity for cellulosic materials [68]. For complete cleaning, it was necessary after tests 1 and 2 (with MS1) to disassemble the plant to be able to clean it more thoroughly (detergent and tap water), since the dye had become embedded in the system and pipes. Regardless of this, the plant and membrane managed to recover after each test. The recovery of the membrane was evidenced by achieving the initial performance of the membrane (Jw and Js), with a recovery average of 94%.

## 4. Conclusions

The results presented in this study demonstrate the potential of the forward osmosis (FO) process to concentrate dyes in textile wastewater for future dye recovery.

A solution of NaCl and reverse osmosis brine from a model seawater desalination solution were tested as draw solutions for the concentration of a textile model solution. Both DSs were effective, but the reverse osmosis brine from a model seawater desalination solution also provided the opportunity to utilize a waste product that is typically difficult to manage. Two different feed solutions were tested, only varying the type of dye. Both feed solutions tested achieved significant color concentration, reaching up to 55%. This was constant regardless of the type of dye (reactive or direct), observing demonstrating that the molecular weight and charge are the main factors that influence the rejection of the dye with the CTA membrane used. Furthermore, the presence of salts in the feed solution was not detrimental to the FO process in this application. In fact, salts play a crucial role in fixing the dye and improving color retention. After all of the tests, the membrane permeability was completely recovered, thus being able to be reused.

Overall, this study provides valuable information on the potential of FO for dye concentration and recovery, offering a promising approach for sustainable wastewater management and resource conservation in the textile industry.

## Figures and Tables

**Table 1:** Dyes used: type, chemical structure and molecular weight (manufacturer data).

Dye	Type	Chemical Structure	MW (g/mol)
Remazol turquoise blue G 133%	Reactive	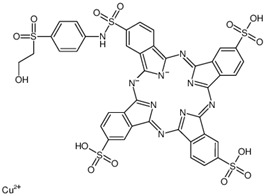	1079.55
Sirius Blue K-CFN	Direct	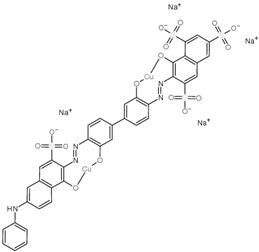	1098.93

**Table 2:** Forward osmosis test for dye concentration study.

Test No.	Nomenclature FS	Composition of FS	Nomenclature DS	Composition of DS
1	MS1	0.05 g·L^−1^ Remazol turquoise blue G 133% + 0.5 g·L^−1^Na_2_SO_4_ + 0.5 g·L^−1^ NaCl	----	1 M NaCl
2	MS1	0.05 g·L^−1^ Remazol turquoise blue G 133% + 0.5 g·L^−1^Na_2_SO_4_ + 0.5 g·L^−1^ NaCl	SBMS	1M NaCl (58.44 g·L^−1^) + 8.50 g·L^−1^ Na_2_SO_4_ + 25.76 g·L^−1^ MgCl_2_·6H_2_O
3	MS2	0.05 g·L^−1^ Sirius Blue K-CFN + 0.5 g·L^−1^ Na_2_SO_4_ + 0.5 g·L^−1^ NaCl	SBMS	1M NaCl (58.44 g·L^−1^) + 8.50 g·L^−1^ Na_2_SO_4_ + 25.76 g·L^−1^ MgCl_2_·6H_2_O

**Table 3:** Characterization of draw solution (DS) and feed solution (FS) before and after test 1 and test 2.

		DS		FS	
Test No.	Parameter	Initial	Final	Initial	Final
1	SO_4_^2−^ (g/L)	-	-	0.46 ± 0.07	0.58 ± 0.10
	Na^+^ (g/L)	30.90 ± 1.14	14.2 ± 0.24	0.28 ± 0.03	0.51 ± 0.07
	Cl^−^ (g/L)	37.75 ± 0.35	20.75 ± 1.35	0.37 ± 0.01	0.66 ± 0.02
	Mg^2+^ (g/L)	0.61 ± 0.01	0.27 ± 0.24	0.01 ± 0.01	0.02 ± 0.02
	Fz	-	-	0.67 ± 0.02	1.03 ± 0.04
	pH	6.46 ± 0.1	6.42 ± 0.1	6.61 ± 0.1	6.72 ± 0.1
2	SO_4_^2−^ (g/L)	5.95 ± 0.07	3.50 ± 0.12	0.43 ± 0.01	0.62 ± 0.03
	Na^+^ (g/L)	32.65 ± 0.21	16.60 ± 0.43	0.30 ± 0.14	0.53 ± 0.07
	Cl^−^ (g/L)	40.00 ± 2.70	27.50 ± 1.03	0.38 ± 0.02	0.71 ± 0.02
	Mg^2+^ (g/L)	2.95± 0.70	1.44 ± 0.20	0.01 ± 0.00	0.03 ± 0.00
	Fz	-	-	0.69 ± 0.03	1.07 ± 0.07
	pH	6.18 ± 0.1	6.28 ± 0.1	6.65 ± 0.1	6.62 ± 0.1

**Table 4:** Conductivity and color (Fz) characterization of draw solution (DS) and feed solution (FS) before and after test 3.

		DS		FS	
Test No.	Parameter	Initial	Final	Initial	Final
3	Conductivity (mS/cm)	92.30	59.10	2.13	3.20
	Fz	-	-	0.838	1.275

## Data Availability

The raw data of this study are not public available due to ongoing researches using a part of the data.

## Supplementary Materials

The following supporting information can be downloaded at: https://www.mdpi.com/article/10.3390/membranes13120911/s1, Figure S1: Permeate fluxes of the different salts tested.
