# Supplementary material for: Feasibility of Forward Osmosis to Recover Textile Dyes Using Single Salts and Multicomponent Draw Solutions
Source: Membranes (Basel). 2023 Dec 18;13(12):911. doi: 10.3390/membranes13120911 (PMC10744723; doi:10.3390/membranes13120911)
Supplement: Supplementary file 1 [file membranes-13-00911-s001.zip › membranes-2718700-supplementary.pdf]

Supplementary material

Feasibility of Forward Osmosis to Recover Textile Dyes Using Single Salts and Multicomponent Draw Solutions

Magdalena Cifuentes-Cabezas[1]*, Laura García-Suarez[2], José Luis Soler-Cabezas[1,3], Beatriz Cuartas-Uribe[1,3], Silvia Álvarez-Blanco[1,3], José Antonio Mendoza-Roca[1,3] and María Cinta Vincent-Vela[1,3]

[1] Research Institute for Industrial, Radiophysical and Environmental Safety (ISIRYM), Universitat Politècnica de València, C/Camino de Vera s/n, 46022 Valencia, Spain

[2] Jeanologia S.L, Ronda de Guglielmo Marconi, 12, 46980 Valencia, Spain

[3] Department of Chemical and Nuclear Engineering, Universitat Politècnica de València, C/Camino de Vera s/n, 46022 Valencia, Spain

* Correspondence: magcica@upv.es

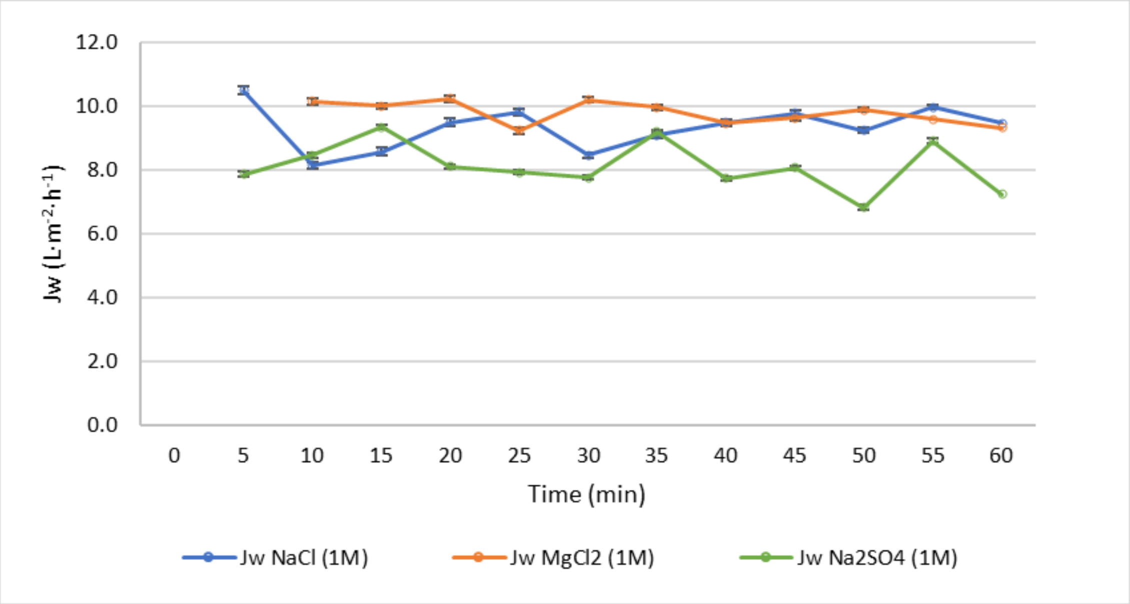

Figure S1. Permeate fluxes of the different salts tested
